# Quality of reporting of randomised controlled trials in chiropractic using the CONSORT checklist

**DOI:** 10.1186/s12998-016-0099-6

**Published:** 2016-06-09

**Authors:** Fay Karpouzis, Rod Bonello, Mario Pribicevic, Allan Kalamir, Benjamin T. Brown

**Affiliations:** PO Box 2108, Rose Bay, Nth 2030 NSW Australia; School of Health Professions, Murdoch University, South St., Murdoch, 6150 WA Australia; Department of Chiropractic, Macquarie University, Balaclava Rd., North Ryde, 2109 NSW Australia

**Keywords:** Manipulation, Chiropractic manipulation, Spinal manipulative therapy, Spine, Musculoskeletal, Quality of reporting, Randomised controlled trials, The CONSORT statement

## Abstract

**Background:**

Reviews indicate that the quality of reporting of randomised controlled trials (RCTs) in the medical literature is less than optimal, poor to moderate, and require improving. However, the reporting quality of chiropractic RCTs is unknown.

As a result, the aim of this study was to assess the reporting quality of chiropractic RCTs and identify factors associated with better reporting quality. We hypothesized that quality of reporting of RCTs was influenced by industry funding, positive findings, larger sample sizes, latter year of publication and publication in non-chiropractic journals.

**Methods:**

RCTs published between 2005 and 2014 were sourced from clinical trial registers, PubMed and the Cochrane Reviews. RCTs were included if they involved high-velocity, low-amplitude (HVLA) spinal and/or extremity manipulation and were conducted by a chiropractor or within a chiropractic department. Data extraction, and reviews were conducted by all authors independently. Disagreements were resolved by consensus. Outcomes: a 39-point overall quality of reporting score checklist was developed based on the CONSORT 2010 and CONSORT for Non-Pharmacological Treatments statements. Four key methodological items, based on allocation concealment, blinding of participants and assessors, and use of intention-to-treat analysis (ITT) were also investigated.

**Results:**

Thirty-five RCTs were included. The overall quality of reporting score ranged between 10 and 33 (median score 26.0; IQR = 8.00). Allocation concealment, blinding of participants and assessors and ITT analysis were reported in 31 (87 %), 16 (46 %), 25 (71 %) and 21 (60 %) of the 35 RCTs respectively. Items most underreported were from the CONSORT for Non-Pharmacological Treatments statement. Multivariate regression analysis, revealed that year of publication (t_32_ = 5.17, *p* = 0.000, 95 % CI: 0.76, 1.76), and sample size (t_32_ = 3.01, *p* = 0.005, 95 % CI: 1.36, 7.02), were the only two factors associated with reporting quality.

**Conclusion:**

The overall quality of reporting RCTs in chiropractic ranged from poor to excellent, improving between 2005 and 2014. This study suggests that quality of reporting, was influenced by year of publication and sample size but not journal type, funding source or outcome positivity. Reporting of some key methodological items and uptake of items from the CONSORT Extension for Non-Pharmacological Treatments items was suboptimal. Future recommendations were made.

**Electronic supplementary material:**

The online version of this article (doi:10.1186/s12998-016-0099-6) contains supplementary material, which is available to authorized users.

## Background

Randomised controlled trials (RCTs) are considered to be the “gold standard” of clinical research [1, 2], by which health care professionals make decisions about the efficacy and effectiveness of interventions [3–6]. However, poorly designed and reported studies continue to be published, leading to a compromised evidence base [7]. This can adversely influence meta-analysis findings and clinical practice recommendations [7–10]. As a result of the poor reporting of RCTs, the CONSORT (Consolidated Standards of Reporting Trials) statement was developed in 1996 [6], and updated in 2010 [11], with the aim of improving the quality of reporting of RCTs through standardization, comprehensiveness and transparency [6, 11].

Reporting research in manual therapies presents obstacles not experienced in medical pharmacological trials. Non-pharmacological trials, such as chiropractic RCTs, test complex therapeutic interventions, which tend to be multi-faceted [12]. As a result, they are more challenging to describe, standardise, reproduce and administer consistently to all participants involved in a clinical trial [12]. These variants, along with others, such as care provider’s expertise may substantially impact estimates of treatment effect [12]. This makes it imperative for such studies to adhere to the CONSORT 2010 [13] and CONSORT for Non-Pharmacologic Treatments statements criteria [12].

Reviews indicate that the quality of reporting of RCTs in the medical literature is less than optimal [14–18]. As a result, many reviewers have drawn conclusions that the overall quality of reporting was poor to moderate [7, 9, 19–21], and require improving [22–26].

To our knowledge there has not been an assessment of the quality of reporting of RCTs in chiropractic. As a result, the aim of this study was to assess the reporting quality of RCTs in chiropractic and to identify factors associated with better reporting quality. The candidate factors that were chosen for this study, have previously been identified in the medical literature as influencing the reporting quality of RCTs [8, 9, 18].

The objectives of this study were to:

1. Assess the overall quality of reporting of RCTs in chiropractic using a customised tool, based on the CONSORT 2010 and CONSORT for Non-Pharmacologic Treatments statements.
2. To report on 4 key methodological items that minimise bias, based on allocation concealment, blinding of participants and assessors, and use of intention-to-treat analysis.
3. To determine factors associated with higher quality of reporting.

We hypothesized that quality of reporting was influenced by industry funding, positive findings, larger sample sizes, latter year of publication and publication in non-chiropractic journals.

## Methods

This study has ethics approval from Murdoch University, Research Ethics and Integrity Office: Ethics #2014/119. The study protocol has been published previously, [27] however an outline is presented below.

### Study selection

**Fig. 1:**
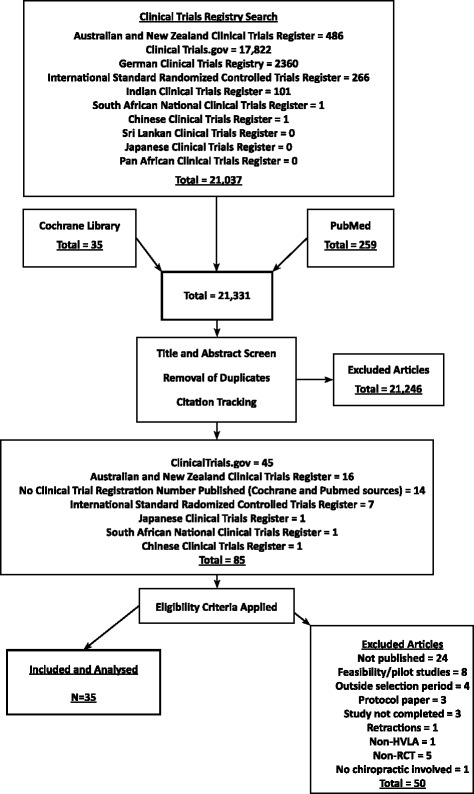
Flow diagram of RCT selection (*N* = *35*)

We searched ten clinical trial registers (refer to Fig. 1) and two electronic databases (PubMed and the Cochrane Database of Systematic Reviews), to identify publications of RCTs involving chiropractic studies, published between January 2005 to July 2014. The search terms used were: “Spine” OR “Lower Extremity” OR “Upper Extremity” AND “Musculoskeletal Manipulations” OR, “Manipulation, Chiropractic” OR “Spinal Manipulative Therapy” AND “Chiropractic”. Full text articles of RCTs in the English language were included if they met inclusion criteria as outlined in Table 1. Article selection and data extraction was conducted by all authors independently, and disagreements were resolved by consensus.

**Table 1:** Inclusion and exclusion criteria

Inclusion Criteria
RCTs with parallel or cross-over study design
Adult study populations with musculoskeletal and non-musculoskeletal with conditions or no condition
Chiropractic high-velocity, low-amplitude (HVLA), musculoskeletal manipulation
Treatment must include chiropractic manipulation, either spinal or peripheral (or both) with/without adjunctive therapy (mobilization, soft tissue therapy, massage, traction, electro-therapies, ultrasound, exercise advice, ergonomic advice, hot/cold therapy, back education)
Comparators: HVLA, placebo, sham treatment or conventional/standard/usual care treatment, or no treatment
Exclusion Criteria
Reviews, systematic reviews and meta-analyses
Non-randomised trial designs (quasi-experimental, observational studies)
Pilot or feasibility studies
Studies with n-of-1
Studies evaluating diagnostic tests, prevention, prognosis, cost-effectiveness, pathophysiological or mechanophysiological mechanisms, validation of questionnaires
Trials not reported as full papers (abstracts), editorials, commentaries, letters, case reports or series, audits, guidelines, historical articles
Methodological/Protocol, epidemiological and qualitative studies
Studies reporting updates of previously published RCTs

We chose to limit this review to high-velocity, low-amplitude (HVLA) studies only, as manual manipulative procedures are the basis of training for all chiropractors. Furthermore, HVLA procedures are reported to be the most popular chiropractic adjusting techniques, used by 93 % of chiropractic practitioners in the US, with similar numbers internationally [28].

It should be recognised that, for the purpose of this study we have included RCTs where both chiropractors and non-chiropractors were involved in the delivery of the interventions, such as physiotherapists, physical therapists and osteopaths [29–32]. However, the HVLA interventions were all delivered by a chiropractor who was part of the study team. We also included studies where the HVLA intervention was the comparator rather than the primary intervention [32–36].

Pilot and feasibility studies were not included as the CONSORT checklist could not be applied to such studies without them being disadvantaged during scoring, in that they typically do not include all items from the CONSORT, such as a power analysis and ITT analysis. Similarly, studies not published as full papers were not included, as it is impossible to properly assess those papers against the CONSORT criteria.

### Review strategy

The characteristics of included studies have been reported in Additional file 1. The characteristics of excluded studies have been reported in Additional file 2.

### Rating the overall reporting quality

This study was modeled upon previously published medical studies assessing the quality of reporting RCTs [9, 14, 16, 18, 19, 37], which used the CONSORT checklists. Furthermore, the CONSORT was used, as it is considered to have both face and content validity and is a measure of methodological quality [38].

A 39-point customised CONSORT checklist was developed by three authors (FK, RB and BB) in order to ascertain the overall quality of reporting of chiropractic RCTs. The overall quality of reporting checklist was developed by integrating items from the CONSORT 2010 [13], and the CONSORT for Non-Pharmacological Treatments statements [12]. Twenty-two items were included from the CONSORT 2010 statement [13], i.e. items one through to 25, excluding items 21, 22 and 24. Items 21 (generalizability [external validity] of the trial findings) and 22 (interpretation of results), which are included in the discussion section, were excluded from the customised checklist because it is challenging to objectively evaluate them [7, 39]. Item 24 (access to trial protocol), was also excluded, as historically it was not a requirement to publish protocols prior to publication of results. Items from the CONSORT 2010 checklist included in our assessment tool are outlined in Table 2. In addition, nine items from the CONSORT for Non-Pharmacological Treatments statement [12], were included i.e. Extensions 1, 3, 4a, 4b, 4c, 8, 13, 15 and ‘New Item’ and are outlined in Table 3.

**Table 2:** Frequencies of CONSORT 2010 items from customized overall quality of reporting checklist (*N* = *35*)

Item	Criterion	CONSORT Description	Total	%
1a	Title	Identification as a randomised trial in the title	26	74
1b	Abstract	Structured summary of trial design, methods, results, and conclusions	35	100
2a	Background	Scientific background and explanation of rationale	35	100
2b		Specific objectives or hypotheses	34	97
3a	Trial Design	Description of trial design (such as parallel, factorial)	18	51
4a	Participants	Eligibility criteria for participants	34	97
4b		settings and locations where the data were collected	17	49
5	Interventions	The interventions for each group with sufficient details to allow replication, including how they were administered	32	91
6a	Outcomes	Completely defined pre-specified primary and secondary outcome measures	32	91
7a	Sample size	How sample size was determined	25	71
8a	Sequence generation	Method used to generate the random allocation sequence	29	83
9	Allocation concealment	Mechanism used to implement the random allocation sequence (such as sequentially numbered containers), describing any steps taken to conceal the sequence until interventions were assigned	31	87
10	Implementation	Was implementation discussed. Who generated the random allocation sequence, who enrolled participants, and who assigned participants to interventions	26	74
11ai	Blinding	Whether or not participants, were blinded to group assignment	16	46
11aii		Whether those assessing the outcomes were blinded to group assignment	25	71
12a	Statistical methods	Statistical methods used to compare groups for outcome(s)	35	100
13a	Participant flow	For each group, the numbers of participants who were randomly assigned, received intended treatment, and were analysed for the primary outcome	29	83
13b		For each group, losses and exclusions after randomization, together with reasons	20	57
14a	Recruitment	Dates defining the periods of recruitment and follow-up	23	66
15	Baseline data	A table showing baseline demographic	32	91
16i	Numbers Analysed	Number of participants (denominator) in each group included in each analysis; state the results in absolute numbers when feasible (e.g., 10/20, not 50 %)	16	46
16ii		“Intention-to-treat” analysis	21	60
17ai	Outcomes and estimation	Primary outcome: a summary of results for each group and the estimated effect size and its precision (e.g., 95 % confidence interval)	26	74
17aii		Secondary outcome: a summary of results for each group and the estimated effect size and its precision (e.g., 95 % confidence interval)	25	71
17b		For binary outcomes, presentation of both absolute and relative effect sizes is recommended	4	11
18	Ancillary Analyses	Results of other analyses performed, including subgroup analyses and adjusted analyses, distinguishing pre-specified	7	20
19	Adverse events	All adverse events or side effects in each intervention group	22	63
20	Limitations	Trial limitations	31	89
23	Registration	Registration number	19	54
25	Funding	Sources of funding and other support	32	91

Legend: Total: Total number of trials reporting item; %: Percentage of trials reporting item

**Table 3:** Frequencies of CONSORT for Non-Pharmacological Treatment statement items from customised overall quality of reporting checklist (*N* = *35*)

Item	Criterion	CONSORT Description	Total	%
1ext	Abstract	Does abstract include-description of the experimental treatment, comparator, care providers, centers, and blinding status	11	31
3ext	Methods	When applicable, eligibility criteria for centers and those performing the interventions (at least one)	13	37
4aext	Interventions	Description of the different components of the interventions and, when applicable, descriptions of the procedure for tailoring the interventions to individual participants	29	83
4bext		Details of how the interventions were standardised (if training was administered)	11	31
4cext		Details of how adherence of care providers with the protocol was assessed or enhanced	1	3
8ext	Randomization	When applicable, how care providers were allocated to each trial group	11	31
13ext	Flow Diagram	The number of care providers or centers performing the intervention in each group and the number of patients treated by each care provider or in each center	3	9
New Item		Details of the experimental treatment and comparator as they were implemented	8	23
15ext	Baseline data	Description of care providers (case volume, qualification, expertise, etc.) and centers	8	23

Legend: Total: Total number of trials reporting item; %: Percentage of trials reporting item; ext: extension criteria from CONSORT for Non-Pharmacological Treatments

The assessment of the adequacy of reporting, was based on the CONSORT 2010 guidelines and its extensions [12, 13, 40]. Items were defined as ‘yes’ if they were clearly and adequately reported and received a score of 1; or ‘no’ if they were unclear or not reported at all, and received a scored of 0. Items that were not applicable to a specific study were defined as ‘not applicable’ (‘N/A’) and were coded 9. The overall quality of reporting score of the trial was calculated as a percentage of the items rated as ‘yes’ (with a score ranging between 0 and 39 points).

Key methodological items, that safeguard against biases [9, 18, 39], have also been reported in the literature [16], such as: allocation concealment (Item 9), blinding (Item 11), and use of ITT analysis (Item 16). The separate assessment of the key methodological items was deemed necessary because, even within published articles with high overall reporting scores, these are often under reported [38] (Table 4). Blinding of participants was scored separately to blinding of assessors. The question of blinding of care-providers was excluded for pragmatic purposes. It has been established that blinding manual therapy practitioners is virtually impossible [41, 42], with similar constraints to the blinding of surgeons in medical clinical trials [14].

**Table 4:** Frequencies of key methodological items from the customised CONSORT checklist (*N* = *35*)

Item No.	Criterion	CONSORT Description	Total	%
9	Allocation concealment	Mechanism used to implement the random allocation sequence (such as sequentially numbered containers), describing any steps taken to conceal the sequence until interventions were assigned	31	87
11ai	Blinding	Whether or not participants, were blinded to group assignment	16	46
11aii	Blinding	Whether those assessing the outcomes were blinded to group assignment	25	71
16ii	Numbers Analysed	“Intention-to-treat” analysis	21	60

Legend: Total: the total number of RCTs that reported this item; %: Percentage of trials reporting item

All authors were involved in the scoring of the RCTs. Each RCT was scored by at least two authors, who were blinded to each other’s results. Results were collated, and any discrepancies were resolved via consensus.

### Definition of trial characteristics

A “positive finding” in a trial was defined as a trial in which the chiropractic intervention was deemed by authors to have statistically significant results and hence was considered superior to the comparator (i.e. placebo/sham, usual care, standard care, medical care, other health care modality, no care or other chiropractic intervention). If the trial produced results that stated that the chiropractic manipulative therapy and the comparator both produced positive outcomes in the study, then the RCT was rated as “no” to the question of “positive finding”, as the chiropractic intervention was not deemed superior to comparator (Refer to Additional file 1).

Trials were considered to be industry-funded, if there was at least partial industry funding. Industry funding included chiropractic research organizations, chiropractic governing bodies or other industry organizations with potentially vested interests in the research. Chiropractic departments funding research within private chiropractic colleges were also deemed to be industry funding, whereas chiropractic and non-chiropractic departments within government educational institutions were considered to be non-industry. Trials that did not have any funding, were also classified as non-industry funding (Refer to Additional file 1).

Trials were considered as published in chiropractic journals, if the journal was dedicated predominantly to the advancement of chiropractic research, education and health care (Refer to Additional file 1).

### Statistical analysis description

This study used descriptive statistics to characterise the overall quality of reporting of chiropractic RCTs, as well as the key methodological items. The percentage of trials that scored ‘yes’ to each CONSORT 2010 item were tabulated and are presented in Table 2. The percentage of trials that scored ‘yes’ to each item from the CONSORT for Non-Pharmacological Treatments, are presented in Table 3. The key methodological items are presented in Table 4.

Two continuous variables were dichotomised. The sample size variable was divided into a smaller group with *n* = 1–100 and a larger group where *n* > 100. The ‘year of publication’ variable was also divided into two time periods (2005–2007 and 2008–2014), which were used in an additional analysis. These time periods were created to distinguish between chiropractic RCTs published before and after the publication of the CONSORT Extension for Non-Pharmacological Treatments statement.

All univariate regression analyses explored associations between the outcome, i.e. the overall quality of reporting score and the exploratory variables (i.e. industry funding, positive findings, sample size group, year of publication and journal type). To test these five exploratory variables, we constructed five univariate models, which included each of the exploratory variables. The exploratory variables that produced results that had a *p* ≤ 0.1, in the univariate regression analysis, were included in the multivariate model [22, 39]. The intention for building this multivariate regression model, was in order to ascertain which of the exploratory variables were independently associated with higher overall quality of reporting scores for the 35 RCTs included in this study. The method used in the multivariate regression analysis was stepwise approach. In the final multivariate regression analysis, variables were considered statistically significant if *p* < 0.05.

An additional ‘final’ multivariable model was created. This model differed in that, the year of publication, which was originally used as a continuous variable, was substituted for the dichotomous variable (as described above). By dividing the year of publication variable into two time periods, pre and post introduction of the CONSORT Extension for Non-Pharmacological Treatments statement, we could analyse the data to investigate whether this new CONSORT statement, impacted the overall quality of reporting.

Variation Inflation Factors (VIFs) were used to test collinearity between exploratory variables. None of the VIFS were >10, indicating that there was no collinearity among the variables. All assumptions for normality and linearity were checked using the Mahalanobis’ and Cook’s Distance statistics. Statistical analyses were conducted using SPSS © 22.0.0.0 (IBM Corporation 2013).

## Results

Sources yielded a total of 21,331 trials. Of the 85 studies that met the first round of inclusion criteria, only 35 (41 %) involving 4435 participants, were published as full-text articles in English journals (Refer to Fig. 1 and Additional file 1). These 35 articles, were assessed for their overall quality of reporting. The RCTs involved adult populations ranging between 17 and 78 years of age. Twenty-five of the 35 (71 %) RCTs reported positive findings in favour of the chiropractic intervention. Seventeen of the 35 (49 %) RCTs were published in a chiropractic journal. Only 43 % (15/35) of the RCTs were industry funded. The sample sizes of the included RCTs ranged between 20 and 444 participants with a mean of 127 (SD ± 102).

### Overall quality of reporting score

The overall quality of reporting score, ranged between 10 and 33 with median score of 26.0 (IQR = 8.00). Individual scores are outlined in the Additional file 1. With regard to reporting frequencies of individual CONSORT items, refer to Tables 2 and 3.

Items that were most poorly reported from the CONSORT 2010 checklist were as follows: item 4b (settings and locations where data were collected), with 17/35 (49 %) of trials reporting this; item 11(a)(i) (whether participants were blinded) with 16/35 (46 %) of trials reporting this; item 13b (the description of each groups losses, exclusions and reasons within the flow diagram), with 20/35 (57 %) reporting on this; item 16 (i) (numbers analysed….in absolute numbers e.g. 10/20, not 50 %) with 16/35 (46 %) of trials reporting this; item 19, (the reporting of adverse events), with 22/35 (63 %) of trials reporting this; and item 23, (the reporting of clinical trial registration) with only 19/35 (54 %) of trials reporting this (Refer to Table 2).

The items that were most underreported, were from the CONSORT Extension for Non-Pharmacological Treatments checklist, with only one of the nine items achieving a high score. Item 4(a) extension requires the reporting of the description of the different components of the interventions and whether they were tailored to individuals, with 29/35 (83 %) of RCTs reporting this item. All other items from the CONSORT for Non-Pharmacological Treatments checklist were very poorly reported with an overall quality of reporting score ranging between 1/35 (3 %) for item 4c extension, (which details how adherence of care providers with the protocol was assessed or enhanced) through to 13/35 (37 %) for item 3 extension, (which describes the eligibility of centers or care providers of the interventions) (Refer to Table 3).

The scoring of the key methodological items also revealed some areas of weakness. Poor reporting of item 11(a)(i) (the blinding of participants) which was reported in 16/35 (46 %) of RCTs, and item 16(ii) (the ITT analysis) which was reported in 21/35 (60 %) of RCTs. The other two items were reported more frequently (Refer to Table 4).

### Results of statistical analyses

**Fig. 2:**
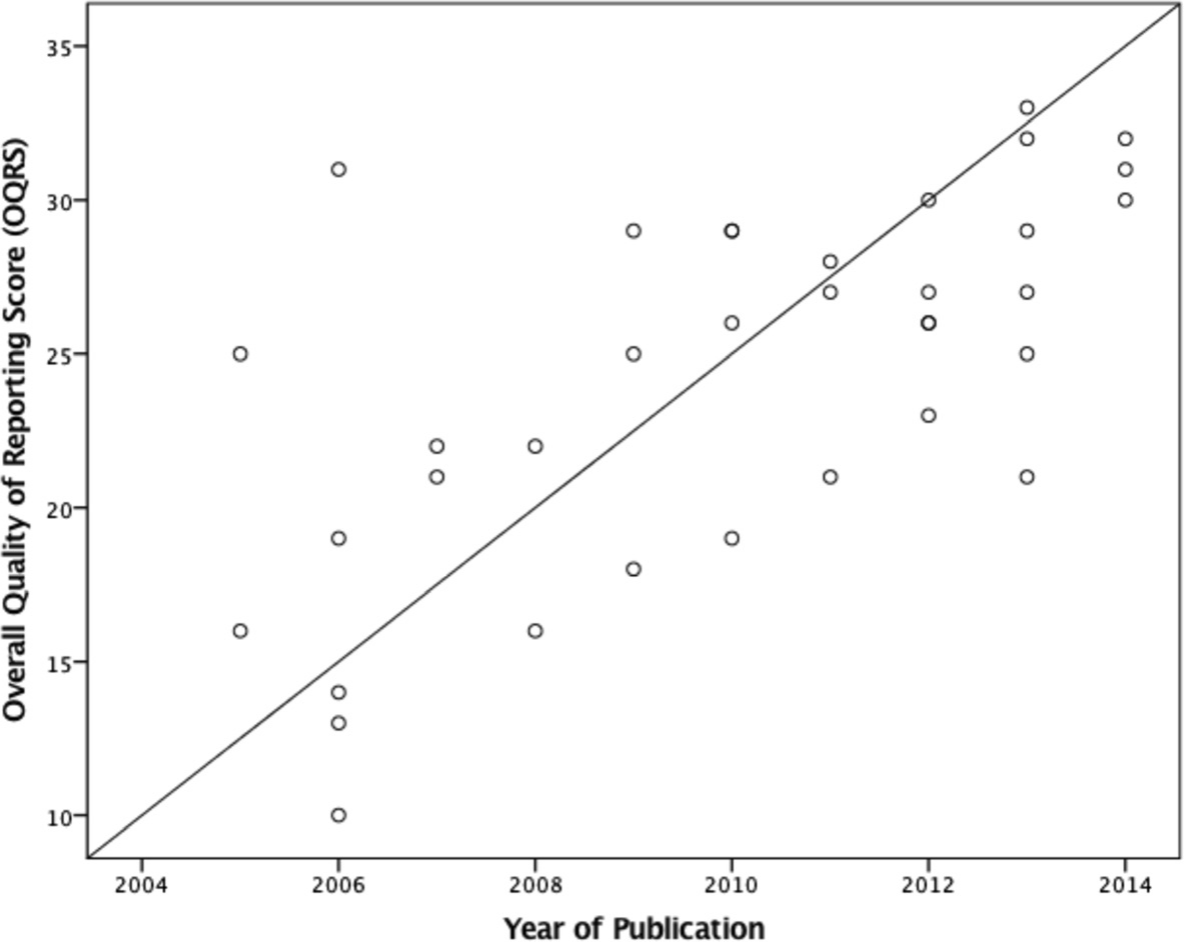
Scatterplot of the correlation between the Overall Quality of Reporting Score and Year of Publication(*N* = *35*)

**Fig. 3:**
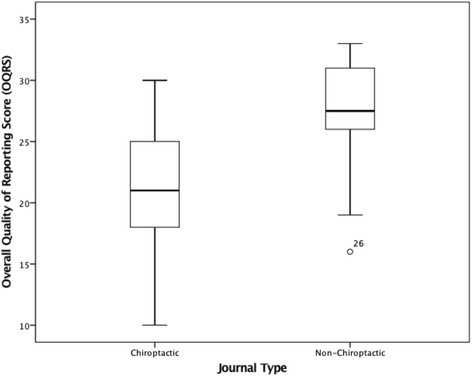
Boxplot of the distribution of Overall Quality of Reporting Scores for Chiropractic vs Non-Chiropractic Journals (*N* = *35*)

**Fig. 4:**
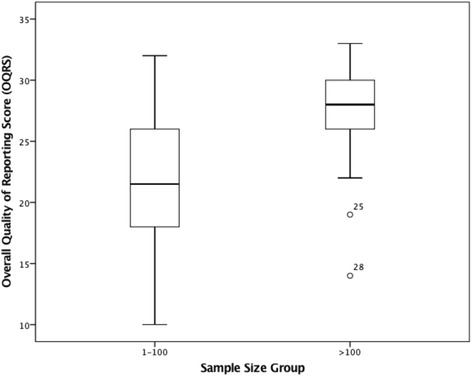
Boxplot of the distribution of Overall Quality of Reporting Scores for Sample Size 1–100 vs >100 (*N* = *35*)

The univariate regression analysis revealed that year of publication (t_33_ = 4.99, *p* = 0.000), journal type (t_33_ = 3.28, *p* = 0.002), and sample size group (t_33_ = 2.75, *p* = 0.010), were all individually and significantly associated with overall quality of reporting (Refer to Table 5 and Figs. 2, 3, and 4 respectively).

**Table 5:** Univariate and multivariate regression analysis for overall quality of reporting score vs exploratory variables (*N* = 35)

Univariate Regression Analysis					
Exploratory Variables	Mean Difference	SE	t	*p*-value	95 % CI
Year of Publication	1.35	0.27	4.99	0.000 ^a^	0.80, 1.90
Journal Type	5.81	1.77	3.28	0.002 ^a^	2.21, 9.42
Sample Size Group	5.05	1.84	2.75	0.010 ^a^	1.31, 8.80
Industry Funding	2.35	2.02	1.16	0.253	−1.76, 6.46
Positive Finding	3.30	2.18	1.51	0.140	−1.14, 7.74
Multivariate Regression Analysis					
Year of Publication (1)	1.26	0.24	5.17	0.000 ^a^	0.76, 1.76
Sample Size Group (1)	4.19	1.39	3.01	0.005 ^a^	1.36, 7.02
Year of Publication Grp (2)	8.16	1.73	4.73	0.000 ^a^	4.64, 11.67
Sample Size Group (2)	4.56	1.45	3.15	0.004 ^a^	1.61, 7.51

Legend: ^a^ statistically significant result; *SE* Standard Error; *t t*-test statistic; *CI* Confidence Interval; *Grp* Group; (1) Multivariate Analysis; (2) Additional analysis

The multivariate regression analysis subsequently revealed that year of publication (t_32_ = 5.17, *p* = 0.000), and sample size group (t_32_ = 3.01, *p* = 0.005), were the only two factors associated with the overall quality of reporting. For each additional year between 2005 and 2014, the overall quality of reporting score increased on average, by an estimated 1.26 points (95 % CI: 0.76, 1.76)(Refer to Table 5). Compared to the smaller sample size group (*n* = 1–100), the larger sample size group (*n* > 100) scored on average 4.19 points higher (95 % CI: 1.36, 7.02) (Refer to Table 5).

**Fig. 5:**
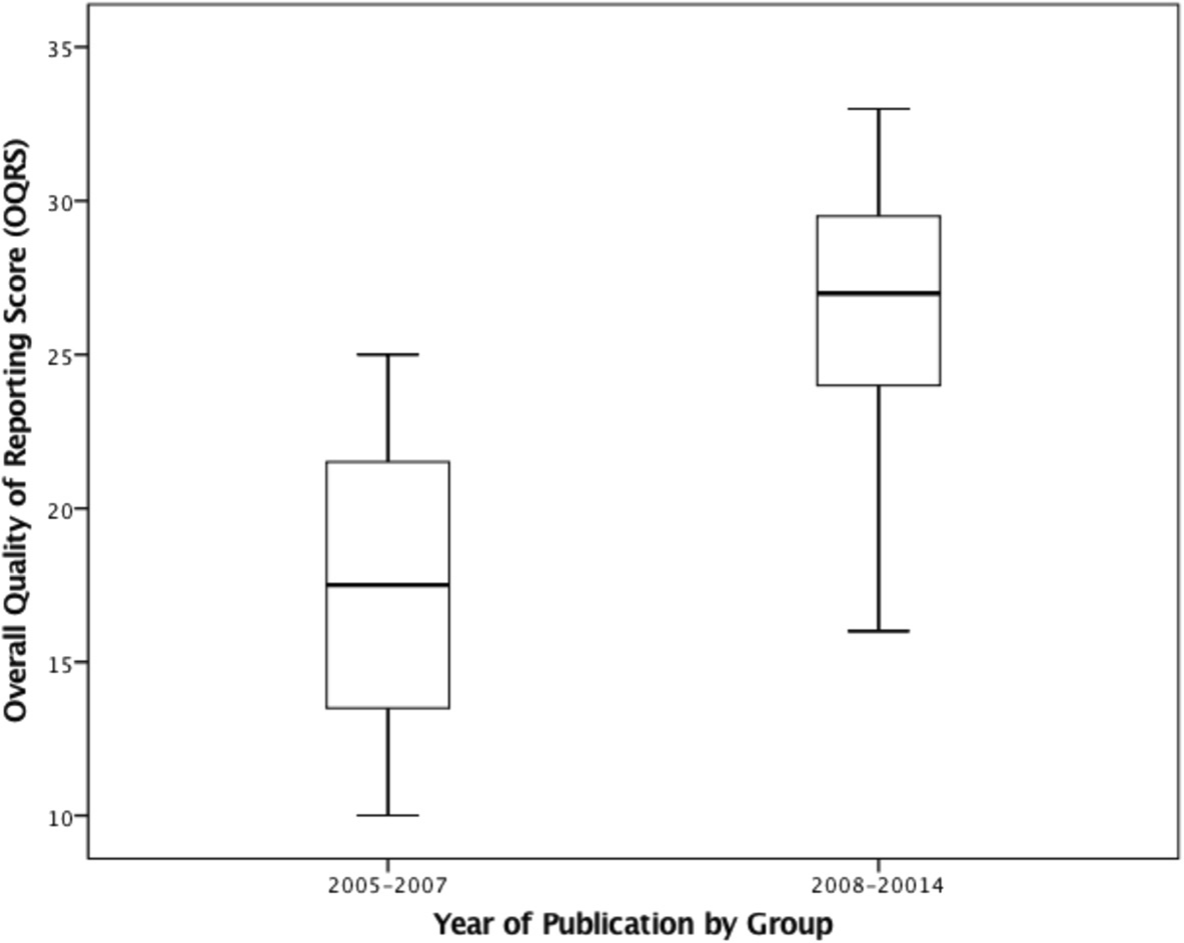
Boxplot of the distribution of Overall Quality of Reporting Scores for Year of Publication by group, 2005–2007 vs 2008–2014 (*N* = *35*)

The additional multivariate regression analysis conducted with the two time periods for the year of publication, revealed that, compared to the period 2005–2007, chiropractic RCTs published between 2008–2014, scored on average 8.16 points higher (95 % CI: 4.64, 11.67) (Refer to Table 5 and Fig. 5). The outcome was not affected by this additional analysis, as the multivariate regression analysis revealed that year of publication and sample size were the only two factors associated with the overall quality of reporting.

The final model in the multivariate regression analysis, revealed that 56 % of the variability in the reporting quality of the included RCTs can be explained by later year of publication and larger sample size (Adjusted *R*^2^ = 0.556).

## Discussion

This appears to be the first study investigating the quality of reporting of chiropractic RCTs relative to the CONSORT checklist. This study suggests, that there has been a significant improvement in the reporting quality of chiropractic RCTs between 2005 and 2014. This may be explained by an increased uptake of the CONSORT guidelines by journal editors and authors, but also by an increasingly professional cadre of chiropractic researchers. Furthermore, studies with sample sizes with *n* > 100, also revealed this trend. This is understandable, as studies with larger sample sizes are associated with greater resources. Furthermore, studies with larger sample sizes are also more likely to be adequately powered in order to find a statistically significant result, if in fact one exists.

While recent publications were more likely to adhere to the CONSORT 2010 criteria, the same cannot be said for the CONSORT Extension for Non-Pharmacological Treatments criteria. Some specific areas, such as: describing items related to care providers and centers, details of adherence to protocols, and how interventions were standardised and if training was administered as prescribed, were very poorly reported. Perhaps this is due to a lack of awareness within the chiropractic research community of these extension criteria.

Under-reported items from the CONSORT 2010 statement included: blinding; explanation of losses and exclusions after randomization with reasons on the flow chart; adverse event reporting; and analysis according to ITT principles, despite the fact these criteria have been established since the 2001 CONSORT statement [40].

Factors such as publishing in non-chiropractic journals showed a trend towards improved quality of reporting scores, although this was not statistically significant in the multivariate regression analysis.

Industry funding was not associated with improved quality of reporting of chiropractic RCTs. In contrast with several medical studies [7, 16, 18, 24, 39, 43], and some reviews [44, 45], which reported concerns that industry funding may be associated with publication bias [44–47].

We also found that a positive finding, was also not associated with the overall quality of reporting within the 35 chiropractic RCTs analysed. This was in contrast to several medical studies that reported that, improved quality of reporting was associated with positive findings [7, 39, 48]. One particular review found that there was a positive association between reporting of favorable outcomes among pharmaceutical trials registered in ClinicalTrials.gov and industry funding [48].

Transparency and accuracy of RCT reporting contributes to the evidence-based information for the profession and will make assessing the validity of RCT results easier. This in turn can lead to better decision-making, helping chiropractic professionals improve their clinical decision making and thus providing better outcomes for patients [49]. As the chiropractic profession is the largest non-medical healthcare profession [50, 51], it is important to continue developing the evidence base so as to inform evidence-based practices. This in turn, will enable the profession to maintain and broaden acceptance from the public, mainstream healthcare and policy makers.

The insights gained from this study should be viewed as an opportunity for improved reporting of RCTs and increased awareness as to the importance of using the CONSORT for Non-Pharmacologic Treatments statement amongst chiropractic researchers. To enhance the practice of evidence-based chiropractic care, researchers are encouraged to implement the CONSORT guidelines with greater rigor, especially in reporting of key methodological items, such as allocation concealment, blinding, and the use of ITT analysis. As these key methodological items can safeguard against bias in the execution and the reporting of future RCTs.

It has been known for some time that the quality of reporting has significantly improved in the medical literature with the adoption of the CONSORT guidelines [10]. Similar outcomes have been reported in a physiotherapy review [52], and a chiropractic review investigating low back and neck pain studies [53]. Our present study suggests that the quality of reporting in chiropractic spinal and non-musculoskeletal studies has also followed this trend.

### Limitations

One limitation to this study was that it is possible that our search strategy did not capture every available chiropractic RCT. We searched ten Clinical Trial registries and two databases and only included published full-text articles in the English language. Additionally, we could not always verify the trial methodology from authors or check their protocols.

Our assessment does not offer any insight into the external validity of the RCTs analysed, as it was too challenging to rate the reporting of such items [7], as there have not yet been any scales developed that have been validated to accomplish this task [39].

Although the CONSORT Extension for Non-Pharmacological Treatments statement was published in 2008 [12], we decided to use time periods starting in 2005, because that was the year the International Committee of Medical Journal Editors published guidelines that required trials to be registered prior to participant enrolment as a precondition for publishing [54]. Furthermore, the original CONSORT was published in 1996 [6], revised in 2001 [55], and again in 2010 [13], and the original CONSORT items continue to exist in all the versions of the CONSORT statements.

Another limitation to the study is that we cannot generalize the results to all forms of chiropractic. We have included studies, which used adjunctive techniques as long as those RCTs also employed an HVLA procedure.

A potential weakness of this study is that, we created a sum score for the overall quality of reporting and used it for both descriptive purposes and as the dependent (outcome) variable for the regression analysis. A problem may be that the attributes we were adding are multi-dimensional and it may not be appropriate to simply add their scores together, as some items in the CONSORT carry greater importance than others. Furthermore, two RCTs may receive the same score but differ in the areas considered deficient with respect to reporting. This can make the overall quality of reporting score somewhat difficult to interpret between studies.

## Conclusion

Reporting quality of RCTs varies widely in chiropractic research. While steady improvement has been observed over the last decade, the chronological improvement observed in this study appears to reflect a more thorough and stringent adoption of the CONSORT criteria. This study suggests that quality reporting was influenced by year of publication and sample size and may also have been be influenced by factors such as journal choice, but not funding source or outcome positivity. This should be regarded as a reassuring finding for the profession and scientific community.

## Recommendations

In light of these findings, we have made some simple recommendations for the improvement of reporting of future chiropractic RCTs.

1. Researchers are encouraged to design and fully report studies to meet the requirements of CONSORT 2010 statement, with extra emphasis on key methodological items:- allocation concealment, blinding of participants and assessors and the use of ITT analysis.
2. Researchers are encouraged to incorporate items from the CONSORT Extension for Non-Pharmacological Treatments statement.
3. Researchers must register their clinical trials, which is in alignment with the standards established by the International Committee of Medical Journal Editors in 2005.
4. Chiropractic journals could exhort researchers to publish the protocols for RCTs in their respective journals for assessment of the study and statistical review, with the understanding that their results are more likely to be published if the protocol meets the CONSORT criteria. (The Lancet is just one of several medical journals that encourages this practice)

## Additional files

Additional file 1:
*Characteristics of the 35 RCTs included in Overall Quality of Reporting Score Analysis*. Legend: Chiro: Chiropractic; JMPT: Journal of Manipulative and Physiological Therapeutics; CJA Chiropractic Journal of Australia; JCCA Journal of the Canadian Chiropractic Association. [29–36, 56–82]. (DOCX 28 kb) Additional file 2:
*Characteristics of the 50 RCTs excluded from Overall Quality of Reporting Analysis*. [83–100]. (DOCX 25 kb)

## Abbreviations

CONSORT: Consolidated Standards of Reporting Trials
HVLA: High-Velocity, Low-Amplitude
ITT: Intention-to-Treat
RCTs: Randomised Controlled Trials
VIFs: Variation Inflation Factors

## References

[1] Cartwright N (2007). Are RCTs the gold standard?. BioSocieties.

[2] Rothwell P (2005). External validity of randomised controlled trials: “To whom do the results apply?”. Lancet.

[3] Keech A, Gebski V, Pike R (2007). Interpreting and reporting clinical trials. A guide to the CONSORT statement and the principles of randomised controlled trials.

[4] Moher D, Jones A, Lepage L, for the CONSORT Group (2001). Use of the CONSORT Statement and quality of reports of randomised trials: A comparative before-and after-evaluation. JAMA.

[5] Altman D, Schulz K, Moher D, Egger M, Davidoff F, Elbourne D, Gotzsche P, Lang T, for the CONSORT Group (2001). The revised CONSORT statement for reporting randomized trials: Explanation and elaboration. Ann Intern Med.

[6] Begg C, Cho M, Eastwood S, Horton R, Moher D, Olkin I, Pitkin R, Rennie D, Schulz K, Simel D (1996). Improving the quality of reporting of randomised controlled trials: the CONSORT statement. JAMA.

[7] Péron J, Pond GR, Gan HK, Chen EX, Almufti R, Maillet D (2012). Quality of reporting of modern randomized controlled trials in medical oncology: a systematic review. J Natl Cancer Inst.

[8] Ntala C, Birmpili P, Worth A, Anderson NH, Sheikh A (2013). The quality of reporting of randomised controlled trials in asthma: Systematic review protocol. Prim Care Respir J.

[9] Borg-Debono V, Zhang S, Ye C, Paul J, Arya A, Hurlburt L, Murthy Y, Thabane L (2012). The quality of reporting of RCTs used within a postoperative pain management meta-analysis, using the CONSORT statement. BMC Anesthesiol.

[10] Plint AC, Moher D, Morrison A, Schulz KF, Altman DG, Hill C, Gaboury I (2006). Does the CONSORT checklist improve the quality of reports of randomised controlled trials? A systematic review. Med J Aust.

[11] The CONSORT 2010 Statement [http://www.consort-statement.org/consort-2010].

[12] Boutron I, Moher D, Altman DG, Schulz KF, Ravaud P, for the CONSORT Group (2008). Extending the CONSORT Statement to randomized trials of nonpharmacologic treatments: Explanation and elaboration. Ann Intern Med.

[13] Schulz KF, Altman DG, Moher D, for the CONSORT Group (2010). CONSORT 2010 Statement: updated guidelines for reporting parallel group randomised trials. Br Med J.

[14] Tiruvoipati R, Balasubramanian SP, Atturu G, Peek GJ, Elbourne D (2006). Improving the quality of reporting randomized controlled trials in cardiothoracic surgery: The way forward. J Thorac Cardiovac Surg.

[15] Partsinevelou A, Zintzaras E (2009). Quality of reporting of randomized controlled trials in polycystic ovary syndrome. Trials.

[16] Chen B, Liu J, Zhang C, Li M (2014). A retrospective survey of quality of reporting on randomized controlled trials of metformin for polycystic ovary syndrome. Trials.

[17] Kim KH, Kang JW, Lee MS, Lee J-D (2014). Assessment of the quality of reporting for treatment components in Cochrane reviews of acupuncture. r Open.

[18] Rios LP, Odueyungbo A, Moitri MO, Rahman MO, Thabane L (2008). Quality of reporting of randomized controlled trials in general endocrinology literature. J Clin Endocr Metab.

[19] Lu L, Luo G, Xiao F (2013). A retrospective survey of the quality of reports and their correlates among randomised controlled trials of immunotherapy for Guillian-Barre syndrome. Immunotherapy.

[20] Bo C, Xue Z, Yoi G, Zelin C, Yang B, Zixu W, Yajun W (2012). Assessing the quality of reports about randomized controlled trials of acupuncture treatment on Diabetic Peripheral Neuropathy. Plos ONE.

[21] Sjögren P, Halling A (2002). Quality of reporting randomised clinical trials in dental and medical research. Br Dent J.

[22] Zhuang L, He J, Zhuang X, Lu L (2014). Quality of reporting on randomized controlled trials of acupuncture for stroke rehabilitation. BMC Compl Alternative Med.

[23] DeMauro SB, Giaccone A, Kirpalani H, Schmidt B (2011). Quality of reporting of neonatal and infant trials in high-impact journals. Pediatrics.

[24] Montori VM, Alonso-Coello P, Wang YG, Bhagra S (2006). Systematic evaluation of the quality of randomized controlled trials in Diabetes. Diabetes Care.

[25] Moberg-Mogren E, Nelson DL (2006). Evaluating the quality of reporting occupational therapy randomized controlled trials by expanding the CONSORT criteria. Am J Occup Ther.

[26] Adetugbo K, Williams H (2000). How well are randomized controlled trials reported in the dermatology literature?. Arch Dermatol.

[27] Karpouzis F, Brown BT, Kalamir A, Pribicevic M, Bonello R (2016). Quality of reporting of randomized controlled trials in chiropractic using the CONSORT checklist: A protocol for a review. Chiropr J Aust.

[28] Christensen MG, Kerkoff D, Kollasch MW. Job Analysis of Chiropractic: A Project Report, Survey Analysis, and Summary of the Practice of Chiropractic within the United States. Greeley, Colo: National Board of Chiropractic Examiners; 2000.

[29] Brennan GP, Fritz JM, Hunter SJ, Thackeray A, Delitto A, Erhard RE (2006). Identifying subgroups of patients with acute/subacute “nonspecific” low back pain: Results of a randomized clinical trial. Spine.

[30] Juni P, Battaglia M, Nuesch E, Hammerle G, Eser P, van Beers R, Vils D, Bernhard J, Ziswiler H-R, Reichenbach S (2008). A randomised controlled trial of spinal manipulative therapy in acute low back pain. Ann Rheum Dis.

[31] Leaver AM, Maher CG, Herbert RD, Latimer J, McAuley JH, Jull G, Refshauge KM (2010). A randomized controlled trial comparing manipulation with mobilization for recent onset neck pain. Arch Phys Med Rehabil.

[32] Eisenberg DM, Post DE, Davis RB, Connelly MT, Legedza ATR, Hrbek AL, Prosser LA, Buring JE, Inui TS, Cherkin DC (2007). Addition of choice of complementary therapies to usual care for acute low back pain: a randomized controlled trial. Spine.

[33] Rosner AL, Conable KM, Edelmann T (2014). Influence of foot orthotics upon duration of effects of spinal manipulation in chronic back pain patients: A randomised clinical trial. J Manipulative Physiol Ther.

[34] Petersen T, Larsen K, Nordsteen J, Olsen S, Fournier G, Jacobsen S (2011). The McKenzie Method compared with manipulation when used adjunctive to information and advice in low back pain patients presenting with centralization or peripheralization: A randomized controlled trial. Spine.

[35] Parkin-Smith GF, Norman IJ, Briggs E, Angier E, Wood TG, Brantingham JW (2012). A structured protocol of evidence-based conservative care compared with usual care for acute nonspecific low back pain: A randomized clinical trial. Arch Phys Med Rehabil.

[36] Poulsen E, Hartvigsen J, Christensen HW, Roos EM, Vach W, Overgaard S (2013). Patient education with or without manual therapy compared to a control grou. in patients with osteoarthritis of the hip. A proof-of- principle three-arm parallel group randomized clinical trial. Osteoarthritis Cartilage.

[37] Piggott M, McGee H, Feuer D (2004). Has CONSORT improved the reporting of randomized controlled trials in the palliative care literature? A systematic review. Palliat Med.

[38] Huwiler-Muntener K, Juni P, Junker C, Egger M (2002). Quality of reporting of randomized trials as a measure of methodologic quality. JAMA.

[39] Lai R, Chu R, Fraumeni M, Thabane L (2006). Quality of randomized controlled trials reporting in the primary treatment of brain tumors. J Clin Oncol.

[40] Moher D, Schulz KF, Altman D, for the CONSORT Group (2001). The CONSORT statement: revised recommendations for improving the quality of reports of parallel-group randomized trials. BMC Med Res Method.

[41] Boutron I, Moher D, Altman D, Schulz K, Ravaud P, for the CONSORT Group (2008). Methods and processes of the CONSORT group: example of an extension for trials assessing nonpharmacologic treatments: explanation and elaboration. Ann Intern Med.

[42] Rubinstein SM, van Middelkoop M, Assendelft WJ, de Boer MR, van Tulder MW: Spinal manipulative therapy for chronic low-back pain. *Cochrane Database of Syst Rev* 2011, Issue 2(Art. No.: CD008112). 10.1002/14651858.CD008112.pub2 PMC12009663 21328304

[43] Bhandari M, Richards RR, Sprague S, Schemitsch EH (2002). The quality of reporting of randomized trials in the journal of bone and joint surgery from 1988 through 2000. J Bone Joint Surg Am.

[44] Lexchin J, Bero LA, Djulbegovic B, Clark O (2003). Pharmaceutical industry sponsorship and research outcome and quality: systematic review. Br Med J.

[45] Okike K, Kocher MS, Mehlman CT, Bhandari M (2008). Industry-sponsored research. Injury.

[46] Djulbegovic B, Lacevic M, Cantor A, Fields KK, Benett CL, Adams JR, Kuderer NM, Lyman GH (2000). The uncertainty principle and industry-sponsored research. Lancet.

[47] Easterbrook PJ, Berlin JA, Gopalan R, Matthews DR (1991). Publication Bias in clinical research. Lancet.

[48] Bourgeois FT, Murthy S, Mandl KD (2010). Outcome reporting among drug trials registered in ClinicalTrials.gov. Ann Intern Med.

[49] Mansholt BA, Stites JS, Derby DC, Boesch RJ, Salsbury SA (2013). Essential literature for the chiropractic profession: a survey of chiropractic research leaders. Chiropr Man Therap.

[50] Coulter ID, Hurwitz EL, Adams AH, Genovese BJ, Hays R, Shekelle PG (2002). Patients using chiropractors in North America: who are they, and why are they in chiropractic care?. Spine (Phila Pa 1976).

[51] Davis MA, Davis AM, Luan J, Weeks WB (2009). The supply and demand of chiropractors in the United States from 1996 to 2005. Altern Ther Health Med.

[52] Moseley AM, Herbert RD, Maher CG, Sherrington C, Elkins MR (2011). Reported quality of randomized controlled trials of physiotherapy interventions has improved over time. J Clin Epidemiol.

[53] Rubinstein SM, van Eekelen R, Oosterhuis T, de Boer MR, Ostelo RWJG, van Tulder MW (2014). The risk of bias and sample size of trials of spinal manipulative therapy for low back and neck pain: analysis and recommendations. J Manipulative Physiol Ther.

[54] DeAngelis C, Drazen J, Frizelle F, Haug C, Hoey J, Horton R, Kotzin S, Laine C, Marusic A, Overbeke A (2004). Clinical trial registration: a statement from the International Committee of Medical Journal Editors. JAMA.

[55] Moher D, Schulz K, Altman D (2001). The CONSORT statement: revised recommendations for improving the quality of reports of parallel-group randomiized trials. JAMA.

[56] Maiers M, Bronfort G, Evans R, Hartvigsen J, Svendsen K, Bracha Y, Schulz C, Schulz K, Grimm R (2014). Spinal manipulative therapy and exercise for seniors with chronic neck pain. Spine J.

[57] Brantingham JW, Parkin-Smith GF, Cassa TK, Globe GA, Globe D, Pollard H, deLuca K, Jensen M, Mayer S, Korporaal C (2012). Full kinetic chain manual and manipulative therapy plus exercise compared with targeted manual and manipulative therapy plus exercise for symptomatic osteoarthritis of the hip: a randomized controlled trial. Arch Phys Med Rehabil.

[58] Haas M, Vavrek D, Peterson D, Polissar N, Neradilek MB (2014). Dose-response and efficacy of spinal manipulation for care of chronic low back pain: a randomized controlled trial. Spine J.

[59] Pollard H, Ward G, Hoskins W, Hardy K (2008). The effect of a manual therapy knee protocol on osteoarthritic knee pain: a randomised controlled trial. J Can Chiropr Assoc.

[60] Hondras MA, Long CR, Cao Y, Rowell RM, Meeker WC (2009). A randomised controlled trial comparing 2 types of spinal manipulation and minimal conservative medical care for adults 55 years and older with subacute or chronic low back pain. J Manipulative Physiol Ther.

[61] Roy RA, Boucher JP, Comtois AS (2009). Heart rate variability modulation after manipulation in pain free patients vs patients in pain. J Manipulative Physiol Ther.

[62] Evans R, Bronfort G, Schulz C, Maiers M, Bracha Y, Svendsen K, Grimm J, Richard , Garvey T, Transfeldt E. Supervised exercise with and without spinal manipulation performs similarly and better than home exercise for chronic neck pain: a randomized controlled trial. Spine. 2012;37(11):903–14. 10.1097/BRS.0b013e31823b3bdf 22024905

[63] Bronfort G, Maiers MJ, Evans RL, Schulz CA, Bracha Y, Svendsen KH, Grimm J, Richard H, Owens JEF, Garvey TA, Transfeldt EE (2011). Supervised exercise, spinal manipulation, and home exercise for chronic low back pain: a randomized clinical trial. Spine J.

[64] McMorland G, Suter E, Casha S, du Plessis SJ, Hurlbert JR (2010). Manipulation or microdiskectomy for sciatica? a prospective randomized clinical study. J Manipulative Physiol Ther.

[65] Srbely JZ, Vernon H, Lee D, Polgar M (2013). Immediate effects of spinal manipulative therapy on regional antinociceptive effects in myofascial tissues in healthy young adults. J Manipulative Physiol Ther.

[66] Walker BF, Hebert J, Stomski NJ, Losco B, French S (2013). Short-term usual chiropractic care for spinal pain: a randomized controlled trial. Spine (Phila PA 1976).

[67] Walker BF, Hebert JJ, Stomski NJ, Clarke BR, Bowden RS, Losco BM, French SD (2013). Outcomes of usual chiropractic. The OUCH randomized controlled trial of adverse events. Spine (Phila PA 1976).

[68] Holt K, Beck R, Sexton S, Taylor HH (2010). Reflex effects of a spinal adjustment on blood pressure. Chiropr J Aust.

[69] Engel RM, Vemulpad SR (2007). The effect of combining spinal manipulation with exercise on the respiratory function of normal individuals: a randomized control trial. J Manipulative Physiol Ther.

[70] Ward J, Coats J, Tyer K, Weigand S, Williams G (2013). Immediate effects of anterior upper thoracic spine manipulation on cardiovascular response. J Manipulative Physiol Ther.

[71] Stochkendahl MJ, Christensen HW, Vach W, Høilund-Carlsen PF, Haghfelt T, Hartvigsen J (2012). Chiropractic treatment vs self-management in patients with acute chest pain: a randomized controlled trial of patients without acute coronary syndrome. J Manipulative Physiol Ther.

[72] Goertz CM, Long CR, Hondras MA, Petri R, Delgado R, Lawrence DJ, Owens JEF, Meeker WC (2013). Adding chiropractic manipulative therapy to standard medical care for patients with acute low back pain. Spine.

[73] Muller R, Giles LG (2005). Long-term follow-up of a randomized clinical trial assessing the efficacy of medication, acupuncture, and spinal manipulation for chronic mechanical spinal pain syndromes. J Manipulative Physiol Ther.

[74] Bishop PB, Quon JA, Fisher CG, Dvorak MFS (2010). The chiropractic hospital-based interventions research outcomes (CHIRO) study: a randomized controlled trial on the effectiveness of clinical practice guidelines in the medical and chiropractic management of patients with acute mechanical low back pain. Spine J.

[75] Shearar KA, Colloca CJ, White HL (2005). A randomized clinical trial of manual versus mechanical force manipulation in the treatment of sacroiliac syndrome. J Manipulative Physiol Ther.

[76] Wilkey A, Gregory M, Byfield D, McCarthy P (2008). A comparison between chiropractic management and pain clinic management for chronic low-back pain in a national health service outpatient clinic. J Altern Complement Med.

[77] Beyerman KL, Palmerino MB, Zohn LE, Kane GM, Foster KA (2006). Efficacy of treating low back pain and dysfunction secondary to osteoarthritis: chiropractic care compared with moist heat alone. J Manipulative Physiol Ther.

[78] Santilli V, Beghi E, Finucci S (2006). Chiropractic manipulation in the treatment of acute back pain and sciatica with disc protrusion: a randomized double-blind clinical trial of active and simulated spinal manipulations. Spine J.

[79] Teodorczyk-lnjeyan JA, Injeyan SH, Ruegg R (2006). Spinal manipulative therapy reduces inflammatory cytokines but not substanc P production in normal subjects. J Manipulative Physiol Ther.

[80] Palmgren PJ, Sandstrom PJ, Lundqvist FJ, Heikkila H (2006). Improvement after chiropractic care in cervicocephalic kinesthetic sensisibility and subjective pain intensity in patients with nontraumatic chronic pain. J Manipulative Physiol Ther.

[81] Saayman L, Hay C, Abrahamse H (2011). Chiropractic manipulative therapy and low-level laser therapy in the management of cervical facet dysfunction: a randomised controlled study. J Manipulative Physiol Ther.

[82] Puhl AA, Injeyan SH (2012). Short-term effects of manipulation to the upper thoracic spine of asymptomatic subjects on plasma concentrations of epinephrine and norepinephrine-a randomized and controlled observational study. J Manipulative Physiol Ther.

[83] Schenk R, Dionne C, Simon C, Johnson R (2012). Effectiveness of mechanical diagnosis and therapy in patients with back pain who meet a clinical prediction rule for spinal manipulation. J Man Manip Ther.

[84] Schulz C, Leininger B, Evans R, Vavrek D, Peterson D, Haas M, Bronfort G (2014). Spinal manipulation and exercise for low back pain in adolescents: study protocol for a randomized controlled trial. Chiropr Man Therap.

[85] Schneider MJ, Brach J, Irrgang JJ, Abbott KV, Wisniewski SR, Delitto A (2010). Mechanical vs manual manipulation for low back pain: an observational cohort study. J Manipulative Physiol Ther.

[86] Miller JE, Newell D, Bolton JE (2012). Efficacy of chiropractic manual therapy on infant colic: A pragmatic single-blind, randomized controlled trial. J Manipulative Physiol Ther.

[87] Goertz CM, Salsbury SA, Vining RD, Long CR, Andresen AA, Jones ME, Lyons KJ, Hondras MA, Killinger LZ, Wolinsky FD (2013). Collaborative care for older adults with low back pain by family medicine physicians and doctors of chiropractic (COCOA): study protocol for a randomized controlled trial. Trials.

[88] Brantingham JW, Globe GA, Cassa TK, Globe D, de Luca K, Pollard H, Jensen ML, Bates C, Jensen M, Mayer S (2010). A single-group pretest posttest design using full kinetic chain manipulative therapy with rehabilitation in the treatment of 18 patients with hip osteoarthritis. J Manipulative Physiol Ther.

[89] Brantingham JW, Globe GA, Jensen ML, Cassa TK, Globe D, Price JL, Mayer SN, Lee FT (2009). A feasibility study comparing two chiropractic protocols in the treatment of patellofemoral pain syndrome. J Manipulative Physiol Ther.

[90] Rowe DE, Feise RJ, Crowther ER, Grod JP, Menke MJ, Goldsmith CH, Stoline MR, Souza TA, Kambach B (2006). Chiropractic manipulation in adolescent idiopathic scoliosis: a pilot study. Chiropr Osteopat.

[91] UK BEAM Trial Team: United Kingdom back pain exercise and manipulation (UK BEAM) randomised trial: effectiveness of physical treatments for back pain in primary care. *BMJ online* 2004. 10.1136/bmj.38282.669225.AE PMC535454 15556955

[92] Thorman P, Dixner A, Sundberg T (2010). Effects of chiropractic care on pain and function in patients with hip osteoarthritis waiting for arthroplasty: a clinical pilot trial. J Manipulative Physiol Ther.

[93] Hawk C, Pfefer MT, Strunk R, Ramcharan M, Uhl N (2007). Feasibility study of short-term effects of chiropractic manipulation on older adults with impaired balance. J Chiropr Med.

[94] Westrom KK, Maiers MJ, Evans RL, Bronfort G (2010). Individualized chiropractic and integrative care for low back pain: the design of a randomized clinical trial using a mixed-methods approach. Trials.

[95] Engel R, Vemulpad SR, Beath K (2013). Short-term effects of a course of manual therapy and exercise in people with moderate chronic obstructive pulmonary disease: a prelimary clinical trial. J Manipulative Physiol Ther.

[96] Botelho MB, Andrade BB (2012). “Effect of cervical spine manipulative therapy on judo athletes’ grip strength”. J Manipulative Physiol Ther.

[97] Strunk RG, Hondras Maria A (2008). A feasibility study assessing manual therapies to different regions of the spine for patients with subacute or chronic neck pain. J Chiropr Med.

[98] Eisenberg DM, Buring JE, Hrbek AL, Davis RB, Connelly MT, Cherkin DC, Levy DB, Cunningham M, O’Connor B, Post DE (2012). A model of integrative care for low-back pain. J Altern Complement Med.

[99] Vavrek D, Haas M, Peterson D (2010). Physical examination and self-reported pain outcomes from a randomized trial on chronic cervicogenic headache. J Manipulative Physiol Ther.

[100] Hurwitz EL, Morgenstern H, Kominski GF, Yu F, Chiang L-M (2006). A randomized trial of chiropractic and medical care for patients with low back pain: eighteen-month follow-up outcomes from UCLA low back pain study. Spine.

