# Erratum to: Quality of reporting of randomised controlled trials in chiropractic using the CONSORT checklist

**DOI:** 10.1186/s12998-016-0120-0

**Published:** 2016-08-17

**Authors:** Fay Karpouzis, Rod Bonello, Mario Pribicevic, Allan Kalamir, Benjamin T. Brown

**Affiliations:** 1 PO Box 2108, Rose Bay Nth, NSW 2030 Australia; 2 School of Health Professions, Murdoch University, South St., Murdoch, WA 6150 Australia; 3 Department of Chiropractic, Macquarie University, Balaclava Rd., North Ryde, NSW 2109 Australia

## Erratum

It has been brought to our attention that the last statement in the Results section of the Abstract of this article [1] is incorrect. This currently reads as: “Multivariate regression analysis, revealed that year of publication (*t*_32_ = 5.17, *p* = 0.000, 95 % CI: 0.76, 1.76), and sample size (*t*_32_ = 3.01, *p* = 0.005, 95 % CI: 1.36, 7.02), were the only two factors associated with reporting quality”. The error is the test statistic for the year of publication. It should be: “Multivariate regression analysis, revealed that year of publication (*t*_32_ = 1.26, *p* = 0.000, 95 % CI: 0.76, 1.76), and sample size (*t*_32_ = 3.01, *p* = 0.005, 95 % CI: 1.36, 7.02), were the only two factors associated with reporting quality”.
