# Supplementary material for: Quality of reporting of randomised controlled trials in chiropractic using the CONSORT checklist
Source: Chiropr Man Therap. 2016 Jun 9;24:19. doi: 10.1186/s12998-016-0099-6 (PMC4899907; doi:10.1186/s12998-016-0099-6)
Supplement: Additional file 1: — Characteristics of the 35 RCTs included in Overall Quality of Reporting Score Analysis. Legend: Chiro: Chiropractic; JMPT: Journal of Manipulative and Physiological Therapeutics; CJA Chiropractic Journal of Australia; JCCA Journal of the Canadian Chiropractic Association. [29–36, 56–82]. (DOCX 28 kb) [file 12998_2016_99_MOESM1_ESM.docx]

Additional File 1:

Additional File 1**:** *Characteristics of the 35 RCTs included in Overall Quality of Reporting Score (OQRS) analysis*

| **Article** | **Author** | **Journal Name** | **Chiro. Journal** | **Year** | **Cohort** | **Sample Size** | **Condition Studied** | **Positive Finding** | **Industry Funding** | **Funding Source** | **OQRS** |
| --- | --- | --- | --- | --- | --- | --- | --- | --- | --- | --- | --- |
| 7 | Maiers et al[56] | The Spine Journal | No | 2014 | Adults | 241 | Chronic Neck Pain | Yes | No | Department of Health and Human Services (HHS) | 31 |
| 14 | Brantingham et al[57] | Archives of Physical Medicine and Rehabilitation | No | 2012 | Adults | 111 | Osteoarthritis of Hip | Yes | Yes | UniHealth Foundation | 27 |
| 20 | Poulsen et al[36] | Osteoarthritis and Cartilage | No | 2013 | Adults | 118 | Osteoarthritis of Hip | No | Yes | Danish Foundation for Chiropractic Research and Postgraduate Education | 33 |
| 21 | Haas et al[58] | The Spine Journal | No | 2014 | Adults | 100 | Chronic Low Back Pain | Yes | No | National Institutes of Health NCCAM | 32 |
| 28 | Pollard et al[59] | JCCA | Yes | 2008 | Adults | 43 | Osteoarthritis of Knee | Yes | No | Received no funding | 22 |
| 32 | Hondras et al[60] | JMPT | Yes | 2009 | Adults | 240 | Subacute or Chronic Low Back Pain | Yes | No | Palmer College of Chiropractic, Department of Health & Human Services | 29 |
| 35 | Roy et al[61] | JMPT | Yes | 2009 | Adults | 51 | Heart rate modulation | Yes | Yes | Univeriste de Quebec | 18 |
| 38 | Evans et al[62] | Spine | No | 2012 | Adults | 270 | Chronic Neck Pain | Yes | No | Northwestern Health Sciences University, Health Resources Services Administration | 26 |
| 39 | Bronfort et al[63] | The Spine Journal | No | 2011 | Adults | 301 | Chronic Low Back Pain | Yes | No | Northwestern Health Sciences University, Health Resources Services Administration | 27 |
| 40 | McMorland et al[64] | JMPT | Yes | 2010 | Adults | 40 | Sciatica secondary to Lumbar Disk Herniation | No | Yes | Foundation for Chiropractic Education and Research | 26 |
| 42 | Rosner et al[33] | JMPT | Yes | 2014 | Adults | 46 | Chronic Back Pain | Yes | Yes | Logan College of Chiropractic & Foot Levelers | 28 |
| 47 | Srbely et al[65] | JMPT | Yes | 2013 | Adults | 36 | Myofascial tissue | Yes | No | Received no funding | 25 |
| 49 | Walker et al[66] | Spine | No | 2013 | Adults | 183 | Adverse Events | Yes | Yes | Chiropractors Registration Board of Victoria, Australia | 32 |
| 50 | Walker et al[67] | Spine | No | 2013 | Adults | 183 | Spinal Pain | No | Yes | Chiropractors Registration Board of Victoria, Australia | 29 |
| 51 | Parkin-Smith et al[35] | Archives of Physical Medicine and Rehabilitation | No | 2012 | Adults | 118 | Acute Non-specific Low Back Pain | No | No | Received no funding | 26 |
| 58 | Holt et al[68] | CJA | Yes | 2010 | Adults | 70 | Blood Pressure | Yes | No | Received no funding | 17 |
| 59 | Engel et al[69] | JMPT | Yes | 2007 | Adults | 20 | Respiratory Function | Yes | No | Macquarie University, Department of Chiropractic | 21 |
| 64 | Ward et al[70] | JMPT | Yes | 2013 | Adults | 36 | Cardiovascular Response | No | Yes | Texas Chiropractic College | 20 |
| 65 | Leaver et al[31] | Archives of Physical Medicine and Rehabilitation | No | 2010 | Adults | 182 | Neck Pain | No | No | National Health and Medical Research Council project grant | 29 |
| 66 | Stochkendahl et al[71] | JMPT | Yes | 2011 | Adults | 115 | Acute Coronary Syndrome | Yes | Yes | Foundation for Chiropractic Research and Post Graduate Education | 30 |
| 68 | Goertz et al [72] | Spine | No | 2013 | Adults | 91 | Acute Low Back Pain | Yes | No | Samueli Institute for Information Biology | 27 |
| 69 | Muller et al [73] | JMPT | Yes | 2005 | Adults | 62 | Spinal Pain Syndrome | Yes | No | QLD State Government Health Department | 25 |
| 73 | Juni et al [30] | Annals of the Rheumatic Diseases | No | 2009 | Adults | 104 | Acute Low Back Pain | No | No | Swiss Society for Manual Therapy; University of Bern; University Hospital Inselspital, Berne | 25 |
| 74 | Bishop et al[74] | The Spine Journal | No | 2010 | Adults | 92 | Acute Mechanical Low Back Pain | Yes | No | University of British Columbia | 29 |
| 76 | Brennan et al[29] | Spine | No | 2006 | Adults | 123 | Acute/Subacute NS Low Back Pain | Yes | No | Deseret Foundation | 19 |
| 78 | Shearar et al[75] | JMPT | Yes | 2005 | Adults | 60 | Sacroiliac Joint Syndrome | Yes | No | None declared | 16 |
| 79 | Wilkey et al [76] | J Alternative and Complementary Medicine | No | 2008 | Adults | 30 | Chronic Low Back Pain | Yes | Yes | Durban institute of Technology Chiropractic Department | 16 |
| 81 | Beyerman et al[77] | JMPT | Yes | 2006 | Adults | 250 | Low Back Pain & Osteoarthritis | Yes | No | Farington Foundation | 14 |
| 86 | Eisenberg et al[32] | Spine | No | 2007 | Adults | 444 | Acute Low Back Pain | Yes | No | Bernard Osher Foundation | 22 |
| 87 | Santilli et al[78] | The Spine Journal | No | 2006 | Adults | 102 | Acute LBP with sciatica and disc protrusion | Yes | No | Centro Studi Patologia Vertebrale | 31 |
| 88 | Teodorczyk-Injeyan et al[79] | JMPT | Yes | 2006 | Adults | 64 | Inflammatory Cytokines | Yes | Yes | Canadian Memorial Chiropractic College | 10 |
| 92 | Petersen et al[34] | Spine | No | 2011 | Adults | 350 | Low Back Pain | No | No | The Danish Rheumatism Association | 28 |
| 95 | Palmgren et al[80] | JMPT | Yes | 2006 | Adults | 41 | Nontraumatic Chronic Neck Pain | Yes | Yes | Scandanavian College of Chiropractic | 12 |
| 97 | Saayman et al[81] | JMPT | Yes | 2011 | Adults | 60 | Cervical Facet Dysfunction | Yes | Yes | The Laser Research Centre, the Department of Chiropractic University of Johannesburg | 21 |
| 99 | Puhl et al[82] | JMPT | Yes | 2012 | Adults | 56 | Norepinephrine and Epinephrine/Thoracic hypomobile joints | No | Yes | Research Division of Canadian Memorial Chiropractic College | 23 |

**Legend:** Chiro: Chiropractic; JMPT: Journal of Manipulative and Physiological Therapeutics; CJA Chiropractic Journal of Australia; JCCA Journal of the Canadian Chiropractic Association.
