# Supplementary material for: Quality of reporting of randomised controlled trials in chiropractic using the CONSORT checklist
Source: Chiropr Man Therap. 2016 Jun 9;24:19. doi: 10.1186/s12998-016-0099-6 (PMC4899907; doi:10.1186/s12998-016-0099-6)
Supplement: Additional file 2: — Characteristics of the 50 RCTs excluded from Overall Quality of Reporting Analysis. [83–100]. (DOCX 25 kb) [file 12998_2016_99_MOESM2_ESM.docx]

**Additional File 2:** *Characteristics of the 50 RCTs excluded from OQRS Analysis*

|  | **Author/Chief Investigator** | **Registry**  **Number** | **Year Registered** | **Reason for Exclusion** |
| --- | --- | --- | --- | --- |
| #1 | Hawk, C. | NCT00163124 | 2005 | Not published by July 2014 |
| #3 | Schenk, R. [83] | NCT00916734 | 2009 | No chiropractors involved in study |
| #4 | Lee, A | NCT00981331 | 2009 | Study not completed |
| #5 | Schulz, G. [84] | NCT01096628 | 2010 | Protocol |
| #6 | Bronfort, G. | NCT00269321 | 2005 | Not published by July 2014 |
| #8 | Bronfort, G. | NCT00494065 | 2007 | Not published by July 2014 |
| #9 | Not Stated | NCT00029770 | 2002 | Study not completed |
| #10 | Bar-Haim, S. | NCT00964717 | 2009 | Not published by July 2014 |
| #11 | Dougherty, P. | NCT00475787 | 2007 | Not published by July 2014 |
| #12 | Schneider, MJ. [85] | NCT01211613 | 2010 | Observational study/Non-RCT |
| #13 | Miller, JE. [86] | NCT01513304 | 2012 | Paediatric study/Non-HVLA |
| #15 | Goertz, C. [87] | NCT01312233 | 2011 | Protocol |
| #16 | Brantingham, J. [88] | NCT01188837 | 2010 | Single group study/ Non-RCT |
| #17 | Pfefer, MT. | NCT00497861 | 2007 | Not published by July 2014 |
| #18 | Brantingham, J. [89] | NCT00401050 | 2009 | Feasibility/pilot study |
| #19 | White, A. | NCT00010621 | 2001 | Trial outside of reference period (Pre-2005) |
| #22 | Dougherty, P. | ISRCTN30511490 | 2013 | Clinical prediction/Non-RCT |
| #23 | Nook, B. | ISRCTN68305337 | 2007 | Not published by July 2014 |
| #24 | Rowe, D. [90] | ISRCTN41221647 | 2006 | Feasibility/pilot study |
| #25 | Coulton, S. [91] | ISRCTN32683578 | 2004 | Trial outside of reference period (Pre-2005) |
| #26 | Humphreys, K. | ISRCTN88585962 | 2012 | Study not completed |
| #27 | Lubbe, D. | NCT01196949 | 2010 | Not published by July 2014 |
| #29 | Enix, D. | NCT02031562 | 2013 | Not published by July 2014 |
| #30 | Goertz, C. | NCT00830596 | 2009 | Not published by July 2014 |
| #31 | Thorman, PM. [92] | NCT01025661 | 2009 | Feasibility/pilot study |
| #33 | Hawk, C. [93] | NCT00497510 | 2007 | Feasibility/pilot study |
| #34 | Enix, D. | NCT02031562 | 2011 | Not published by July 2014 |
| #36 | Westrom, KK. [94] | NCT00567333 | 2007 | Protocol paper |
| #37 | Wedderkopp, N. | NCT01504698 | 2012 | No published by July 2104 |
| #41 | Giggey, KJ. | NCT00335426 | 2006 | Non-RCT |
| #43 | Malmquist, S. | NCT01098136 | 2010 | Not published by July 2014 |
| #44 | Larsen, JP. | NCT00974103 | 2009 | Not published by July 2014 |
| #45 | Eisenberg, D. | NCT00010985 | 2003 | Trial outside of reference period (Pre-2005) |
| #46 | Björndahl, L. | ISRCTN49222271 | 2007 | Not published by July 2014 |
| #48 | Pollard, H. | ACTRN12611000681954 | 2008 | Not published by July 2014 |
| #52 | Pollard, H. | ACTRN12609000664246 | 2009 | Not published by July 2014 |
| #53 | Pribicevic, M. | ACTRN12609000400268 | 2009 | Not published by July 2014 |
| #54 | Holt, K. | ACTRN12608000333314 | 2008 | Not published by July 2014 |
| #55 | Holt, K. | ACTRN12607000509460 | 2007 | Not published by July 2014 |
| #56 | Engel, R. | ACTRN12607000388415 | 2007 | Not published by July 2014 |
| #57 | Hayek, R. | ACTRN12607000270415 | 2007 | Not published by July 2014 |
| #60 | Engel, R. [95] | ACTRN12606000369527 | 2006 | Feasibility/pilot study |
| #61 | Hoskins, W. | ACTRN12608000533392 | 2008 | Study Retracted |
| #62 | Zhang, C. | ChiCTR-TRC-14004555 | 2005 | Not published by July 2014 |
| #63 | Botelho, MB. [96] | ISRCTN38228413 | 2009 | Feasibility/pilot study |
| #90 | Strunk, RG. [97] | -- | 2008 | Feasibility/pilot study |
| #93 | Eisenberg, D. [98] | NCT00065975 | 2012 | Feasibility/pilot study |
| #94 | Vavrek, D. [99] | NCT00246350 | 2005 | Diagnostic study/ Non-RCT |
| #96 | Petersen, CD. | NCT00937365 | 2009 | Not published by July 2014 |
| #98 | Hurwitz, EL. [100] | -- | 2006 | Trial completed outside of reference period (Pre-2005) |
